# Superimposed traumatic brain injury modulates vasomotor responses in third-order vessels after hemorrhagic shock

**DOI:** 10.1186/1757-7241-21-77

**Published:** 2013-11-21

**Authors:** Bo Chen, Manuel Mutschler, Yongjun Yuan, Edmund Neugebauer, Qiaobing Huang, Marc Maegele

**Affiliations:** 1 Department of Pathophysiology, Key Laboratory for Shock and Microcirculation Research, Southern Medical University (SMU), Tong He, 510515 Guangzhou, People's Republic of China; 2 Institute for Research in Operative Medicine (IFOM), Private University of Witten-Herdecke, Cologne Merheim Medical Center (CMMC), Ostmerheimerstr. 200, D-51109 Cologne, Germany; 3 Department of Traumatology, Orthopedic Surgery and Sportsmedicine, Private University of Witten-Herdecke, Cologne-Merheim Medical Center (CMMC), Ostmerheimerstr. 200, D-51109 Cologne, Germany

**Keywords:** Trauma, Hemorrhagic shock, Lateral fluid percussion, Brain injury, Spinotrapezius muscle, Third order vessels, Vasomotor response, Microcirculation

## Abstract

**Background:**

Traumatic brain injury (TBI) and hemorrhagic shock (HS) are the leading causes of death in trauma. Recent studies suggest that TBI may influence physiological responses to acute blood loss. This study was designed to assess to what extent superimposed TBI may modulate physiologic vasomotor responses in third-order blood vessels in the context of HS.

**Methods:**

We have combined two established experimental models of pressure-controlled hemorrhagic shock (HS; MAP 50 mmHg/60 min) and TBI (lateral fluid percussion (LFP)) to assess vasomotor responses and microcirculatory changes in third-order vessels by intravital microscopy in a spinotrapezius muscle preparation. 23 male Sprague–Dawley rats (260–320 g) were randomly assigned to experimental groups: i) Sham, ii) HS, iii) TBI + HS, subjected to impact or sham operation, and assessed.

**Results:**

HS led to a significant decrease in arteriolar diameters by 20% to baseline (p < 0.01). In TBI + HS this vasoconstriction was less pronounced (5%, non-significant). At completed and at 60 minutes of resuscitation arteriolar diameters had recovered to pre-injury baseline values. Assessment of venular diameters revealed similar results. Arteriolar and venular RBC velocity and blood flow decreased sharply to < 20% of baseline in HS and TBI + HS (p < 0.01). Immediately after and at 60 minutes of resuscitation, an overshoot in arterial RBC velocity (140% of baseline) and blood flow (134.2%) was observed in TBI + HS.

**Conclusion:**

Superimposed TBI modulated arteriolar and venular responses to HS in third-order vessels in a spinotrapezius muscle preparation. Further research is necessary to precisely define the role of TBI on the microcirculation in tissues vulnerable to HS.

## Background

Traumatic brain injury (TBI) and uncontrolled hemorrhage are the leading causes of death in multiple injured trauma patients [1-4] and the coincidence of TBI and hemorrhage after trauma has been associated with an even worse overall morbidity and mortality [5-7]. Several studies have shown that superimposed TBI to hemorrhage, e.g. the addition of a traumatic impact to the brain on top of hemorrhage, may result in impaired cardiovascular compensation [8-12] and a reduced ability to modulate vascular tone [13]. More recent studies have demonstrated disturbances in the autonomic response to hemorrhage if TBI is present [8] and that TBI may attenuate or delay the physiological bradycardic and hypotensive response to acute extracranial blood loss [9]. Additionally, the presence of TBI has been associated with an early increase in baroreflex sensitivity, another key component of cardiovascular hemostasis [10].

In order to maintain tissue perfusion and to preserve physiological hemodynamics, an adequate function of the peripheral microcirculation within the different organ systems is fundamental [14]. Extracranial hemorrhage is known to be associated with microcirculatory hypoperfusion and subsequent tissue damage by inducing vasoconstriction and decreasing arterial blood flow [15-17]. However, this microcirculatory response to extracranial hemorrhage when coincidenced with a superimposed TBI, as frequently encountered in the clinical setting, is yet to be defined. Apart from directly inflicting and damaging the brain, experimental TBI itself has also been shown affect systemic circulation. Several studies have demonstrated that various models of TBI may create an acute and transient systemic hypertension and bradycardia which is followed by a hypodynamic state with heterogeneous hypoperfusion among organs at increasing magnitudes of injury [18,19].

In the present study, we have combined two well-established and characterized experimental models, i.e. i.) a pressure-controlled hemorrhagic shock model with ii.) a standardized and clinically relevant model of experimental TBI, to assess vasomotor responses and microcirculatory changes in third-order arterioles and venules of a spinotrapezius muscle preparation by using intravital microscopy.

## Methods

### Animals

Male Sprague–Dawley rats (n = 23; body weight 260–320 grams) were housed individually under controlled environmental conditions with a 12-hours light/dark cycle and with ad libitum access to pellet food and water throughout the study. The animals were purchased from the Experimental Animal Center of the Southern Medical University (Guangzhou, P. R. China (Certification: SCXK (Guangdong) 20060015)). The sample size per group was calculated based upon previous work and experience to statistically test for differences in neurofunction/-behaviour 24 hours and seven days post-injury at an overall error level of 0.05 [20,21]. All surgical interventions, i.e. pressure-controlled hemorrhagic shock, TBI via fluid-percussion as well as intravital microscopy, were performed under deep anaesthesia with sodium pentobarbital (60 mg/kg/body weight i.p.) using a standardized protocol established in our laboratory. All experiments were conducted in the Key Laboratory of Shock and Microcirculation (Southern Medical University, Guangzhou, P.R. China), were in accordance with the Chinese National Guidelines for the Use and Care of Experimental Animals and were approved by the Institutional Experimental Animal Ethics Committee of the Southern Medical University, Guangzhou (P.R. China). The Chinese animal guidelines adhere to the ARRIVE (Animal Research: Reporting *In Vivo* Experiments) guidelines which are intended to improve the reporting of animal experiments. All efforts were made to reduce the number of animals used and to minimize animal discomfort. All animals received postoperative oral pain medication for 3 days post intervention (Tramadol 2,5 mg/100 ml in drinking water).

### Surgical preparation and pressure-controlled hemorrhagic shock

Pressure-controlled hemorrhagic shock was inflicted as previously described by our group [22,23]. Briefly, both femoral arteries and the left femoral vein were cannulated using polyethylene cannulaes for continuous monitoring of arterial blood pressure, blood withdrawal, and resuscitation. To inflict hemorrhagic shock with a target mean arterial blood pressure (MAP) of 50 mmHg, blood was withdrawn from the arterial catheter within 10 minutes under continuous blood pressure monitoring. For anticoagulation, the withdrawn blood was mixed with 12.5 IU/ml heparin immediately and stored for later resuscitation at the end of the experimental phase.

### Lateral fluid percussion brain injury (LFP)

The lateral fluid-percussion (LFP) brain injury is one of the most widely used and well characterized models of experimental traumatic brain injury and has been described in detail elsewhere [24-28]. Briefly, anesthetized rats were placed in a stereotaxic frame. After incision of the scalp, the temporal muscles were reflected and a 4.8 mm craniotomy was drilled (2.5 mm lateral to the sagital sinus and centered between bregma and lambda), keeping the dura mater intact. A hollow female Luer-Lok fitting was placed directly over the dura and rigidly fixed using dental cement. Prior to the induction of trauma, the female Luer-Lok was connected to the fluid percussion injury device via a transducer (Bimedical Engineering Facility, Medical College of Virginia, USA), creating a fluid-filled system in connection with the dura. For the infliction of TBI, a metal pendulum was released from a pre-selected height leading to a rapid injection of normal saline into the closed cranial cavity. A pulse of increased intracranial pressure of 21 to 23 ms duration was created as well as controlled and recorded by an oscilloscope (Agilent 54622D, MEGAZoom, Germany). The severity of injury inflicted on the animal can be adjusted by the amount of force generated by the pendulum. For the present experiment an injury level of moderate severity was induced (2.5 ± 0.2 atmospheres [20,21]). Hemorrhagic shock only and Sham animals underwent identical preparatory procedures including craniotomy but were not injured.

### Histopathological changes after LFP

**Figure 1:**
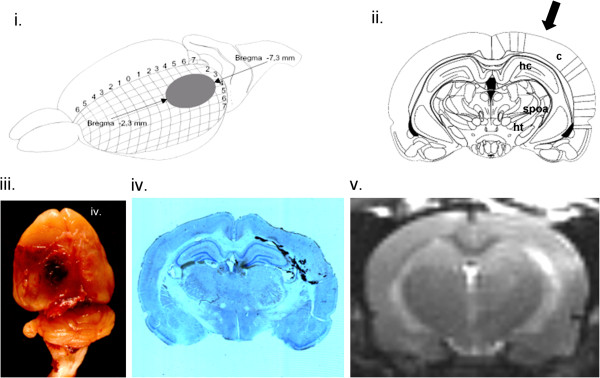
**Traumatic brain injury via lateral fluid-percussion (LFP).** The lesion inflicted via LFP is typically located between Bregma −2,3 mm and −7,3 mm **(i.)**. Injured regions include the ipsilateral cortex (c) but also deeper brain/brain stem structures such as hippocampal (hc), thalamic, hypothalamic (ht), pontine, and supraoptic areas (spoa) (**ii.**; coronar section of the brain at Bregma −4,52; arrow indicates direction of impact). The model used for this experiment reproducible creates a subdural haematoma **(iii.)**, focal cortical contusion ipsilateral to the injured site of the brain with a characteristic pattern of haemorrhage involving subcortical white matter, adjunct cortex, subarachnoid space, hippocampus as well as deep brain structures such as thalamus, hypothalamus and pontine regions (**iv.**; Nissl staining), and a mass shift towards the contralateral hemisphere as a consequence of the trauma-induced ipsilateral cytotoxic/vasogenic edema (**v.**; T-2-weighted MRI). (modified from [31]; see also “The Rat Brain in Stereotaxic Coordinates” by Praxinos and Watson; Academic Press, 1998).

The temporal and spatial histopathological consequences of the LFP model have been intensively investigated and reported [24-35]. In principle, the model reflects a clinical contusion without skull fracture with a direct relationship between pathological alteration and the magnitude of injury inflicted. The model established by our group reproducibly creates a focal cortical contusion ipsilateral to the injured site of the brain accompanied by a characteristic pattern of hemorrhage and a mass shift towards the contralateral hemisphere as a consequence of the trauma-induced ipsilateral cytotoxic and/or vasogenic brain edema [31]. Despite the injury is inflicted unilaterally, bilateral diffuse white matter damage is induced remote from the injury site [34] and severity-graded injury of the brain-stem may account for the high morbidity in this model compared to others [24,29-31,35]. The delayed progression of brain damage in this model is accompanied by astrocytosis, diffuse axonal injury, inflammatory events, cortical spreading depression and neurodegeneration [24,28,29,33,34]. Figure 1 displays an overview of some of the relevant histopathological consequences inflicted by the LFP model. The LFP model for TBI was chosen since it is considered as one of the most clinically relevant experimental models of TBI in rodents and short- as well as long-term behavioural, physiological and histological consequences have been extensively characterized and the injuries are highly reproducible once the model is locally established [24-35]. To date, the lateral fluid percussion brain injury model is the primary model used for experimental research in neurotrauma mechanistic studies and drug screening trials [30]. It is certainly accepted that no single animal model of TBI can accurately reproduce the complete pathophysiological complexities of human TBI [30].

### Microcirculation: preparation of the spinotrapezius muscle and intravital microscopy

In order to assess vasomotor responses and microcirculatory changes to hemorrhagic shock with or without superimposed TBI intravital microscopy of the spinotrapezius muscle was performed. The spinotrapezius muscle locates anatomically in the mid-dorsal region where the muscle originates in the lower thoracic and upper lumbar region and inserts onto the spine of the scapula. The preparation of the spinotrapezius muscle was performed as previously described by Gray [36]. Briefly, the exteriorization of the spinotrapezius muscle was performed with marginal damage to the fascia, only. No evidence of local trauma which may impact regional blood flow by this model has been reported yet. Throughout the surgical preparation and the experimental course, the exposed tissue was continuously superfused with a heated Krebs-Henseleit bicarbonate-buffered solution to maintain a constant 37°C environment temperature, humidity, pH, and ionic strength of the sample. The solution was heated because otherwise there would have been a loss of heat in the way of perfusion. The exposed spinotrapezius muscle was fixed at six equidistant positions around the caudal periphery to ensure consistent shape and length of the selected vessels. Unbranched, third-order arterioles and venules (diameter range 20–60 μm) were selected at random and videotaped with 2000 frames/s using a CR5000X2 high-speed camera (Optronis, Germany). The vessel diameters and erythrocyte velocities (V_RBC_) were assessed at a rate of 25 frames/s [37,38]. Assuming cylindrical geometry, blood flow was calculated using the formula as previously described [39]: π* Vmean*(D/2)^2^; Vmean = V_RBC_/1.6 and D = diameter of the vessel.

### Experimental protocol

**Figure 2:**
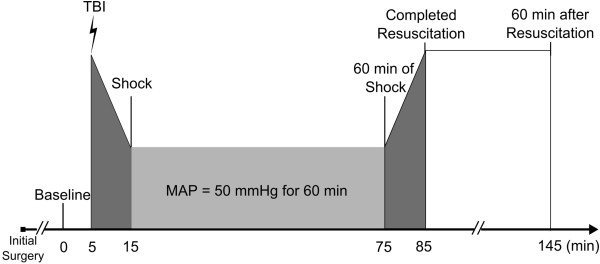
Experimental protocol and flow of experiments.

**Figure 3:**
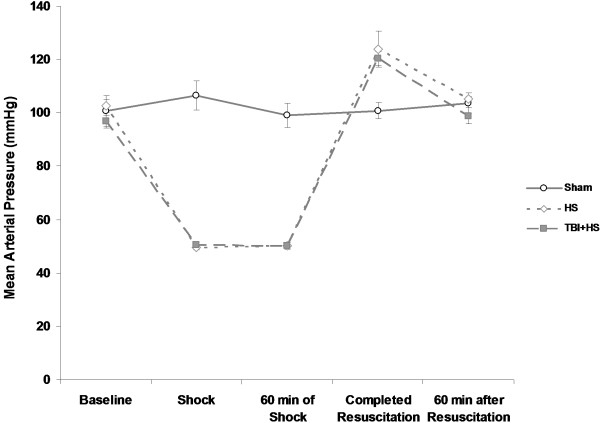
Mean arterial blood pressure (mmHg) during the experiment for the three groups: Sham, hemorrhagic shock (HS) and HS with traumatic brain injury (TBI).

Twenty-three rats were randomly assigned to one of three experimental groups: control (Sham; n = 5), hemorrhagic shock (HS, n = 9), and combined HS and traumatic brain injury (HS + TBI, n = 9). Figure 2 provides an overview of the flow of the experiment. After initial preparation and prior to infliction of the impacts animals were allowed 15 minutes for cardiovascular stabilization followed by baseline measurements for blood pressure and microcirculation. Next, traumatic brain injury via lateral fluid-percussion was induced in the TBI + HS group, followed by pressure-controlled hemorrhagic shock in the HS and TBI + HS groups. The target mean arterial pressure of 50 mmHg was achieved within 10 minutes in both groups and maintained for 60 minutes followed by reinfusion of the shed blood to resuscitate the animals to their pre-injury pressure levels (Figures 2 and 3). Both impacts followed protocols previously established in our laboratory [22,23,31]. Vasomotor responses and microcirculatory changes were assessed by intravital microscopy of the spinotrapezius muscle prior to injury, after reaching the target MAP, after 60 minutes of pressure-controlled hemorrhagic shock as well as after resuscitation and 60 min after resuscitation as depicted in Figure 2. The surviving animals were monitored for pre-defined seven days after injury and then re-anesthetized as previously done for model induction (see above) and sacrificed without any discomfort.

### Statistical analysis

All data are expressed as means ± SEM. Microcirculatory variables are expressed as percent change from baseline. For the comparison of removed blood volumes, the student’s *t*-test was used. To compare the differences between the three groups (Sham, HS, TBI + HS) at different time points, analysis of variance (ANOVA) for repeated measurements was used. The level of statistical significance was set at *P* < 0.05. All data were analyzed using IBM SPSS software (IBM SPSS 19, Chicago, USA).

## Results

### Mean arterial blood pressure (MAP) and mortality

All animals included in this study were comparable at baseline for size, weight, haemoglobin and pH values (data not shown). According to the experimental protocol, a target MAP of 50 mmHg was successfully achieved in all animals (HS only and TBI + HS) within 10 minutes and maintained for 60 minutes of the experiment. In order to achieve this target pressure, comparable blood volumes were withdrawn from both groups (HS only 24.6 ± 1.1 ml; TBI + HS 26.1 ± 1.6 ml). There were no differences in MAP curves observed between HS only and TBI + HS groups (Figure 3). Likewise, between the two different experimental groups, there were no differences in Hb, pH and PaCO_2_ observed upon repeated arterial blood gas analyses (ABG) at baseline and during the entire experimental (data not shown). Superimposed TBI to hemorrhagic shock resulted in a higher early and overall mortality compared to HS alone. While all Sham animals survived the pre-defined study period of seven days, 1/9 animals in the HS group and 4/9 animals in the TBI + HS group died within 24 hours after trauma and within the later course of the experiment (3/9 in HS only and 2/9 in TBI + HS).

### Third-order vessel diameters

**Figure 4:**
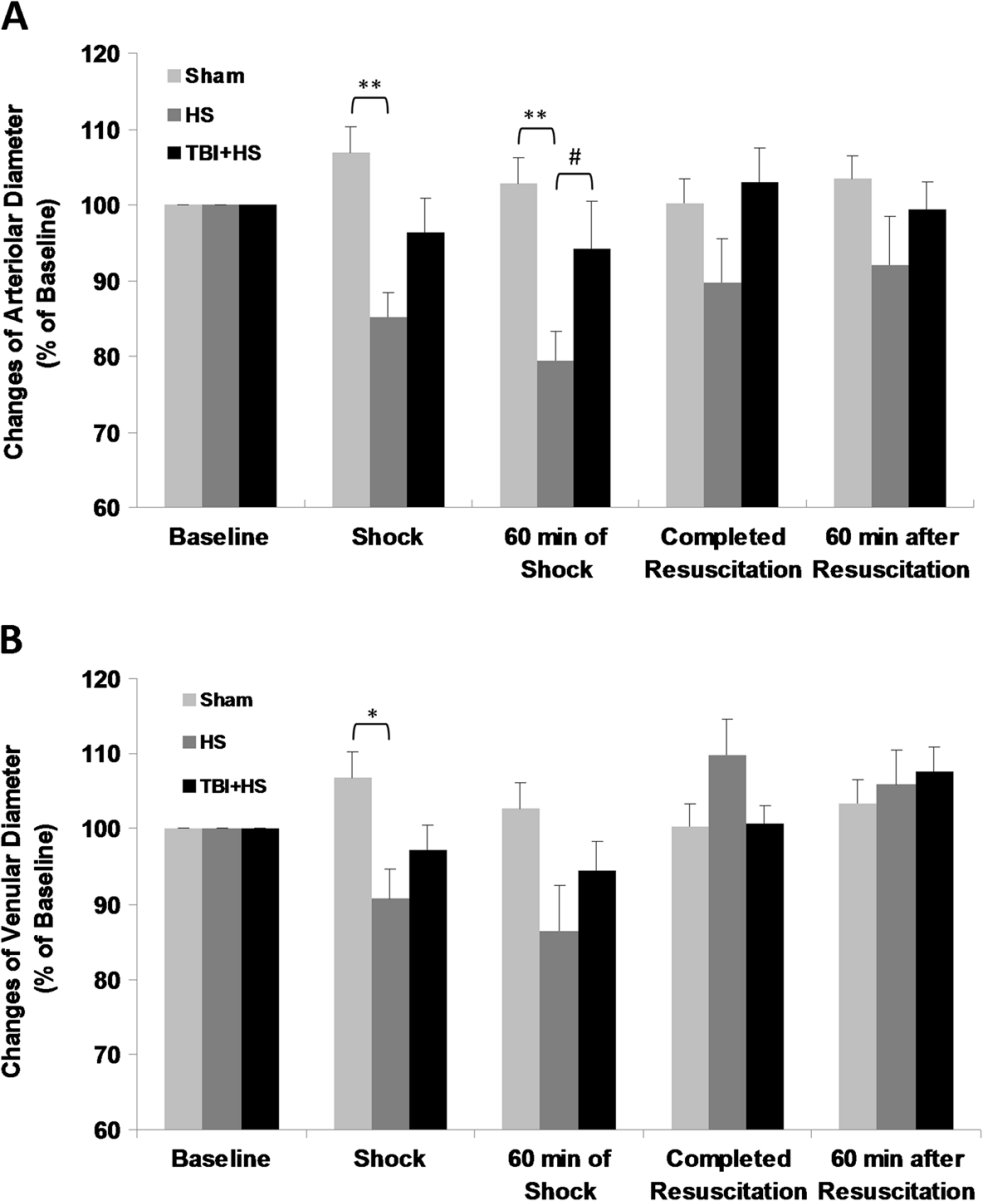
**Vasomotor responses to hemorrhagic shock with and without superimposed TBI by change in arteriolar (A) and venular (B) diameters for the five observation time points.** (* p < 0.05; ** p < 0.01; # p < 0.05).

**Figure 5:**
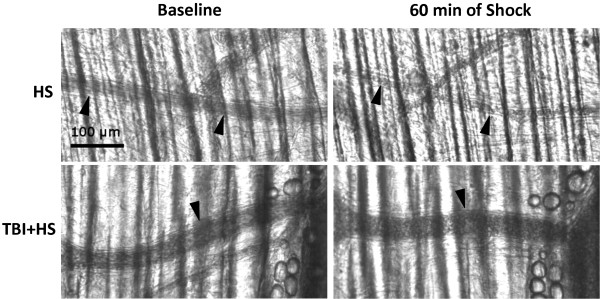
Pictorial example of arteriolar diameter change in response to hemorrhagic shock only (upper panel) and when combined with superimposed TBI (lower panel) at baseline and at 60 minutes of shock.

Pressure-controlled hemorrhage was associated with a significant and progressive decrease in arteriolar diameters (ADs) of third-order vessels in this preparation by 20% to baseline (p < 0.01; Figure 4A). Resuscitation reversed this decrease and at 60 minutes of completed resuscitation HS only animals had regained 90% of their initial ADs at baseline. Combined animals with pressure-controlled hemorrhagic shock and superimposed TBI (TBI + HS) showed a similar pattern of decreased ADs, but by far less pronounced (non-significant; Figure 4A). Furthermore, at completed as well as 60 minutes of resuscitation ADs in these animals had gained pre-injury baseline values. The assessment of venular diameters (VDs) revealed similar results but in general a less distinct decline as opposed to arterioles (Figure 4B). Venular diameters in both groups had recovered to pre-injury baseline values at completed and 60 minutes of resuscitation. Figure 5 shows a pictorial example of the arteriolar diameter change in response to pressure-controlled hemorrhagic shock with and without superimposed TBI at baseline and at 60 minutes of shock.

### Red-blood-cell (RBC) velocity and blood flow in third-order vessels

**Figure 6:**
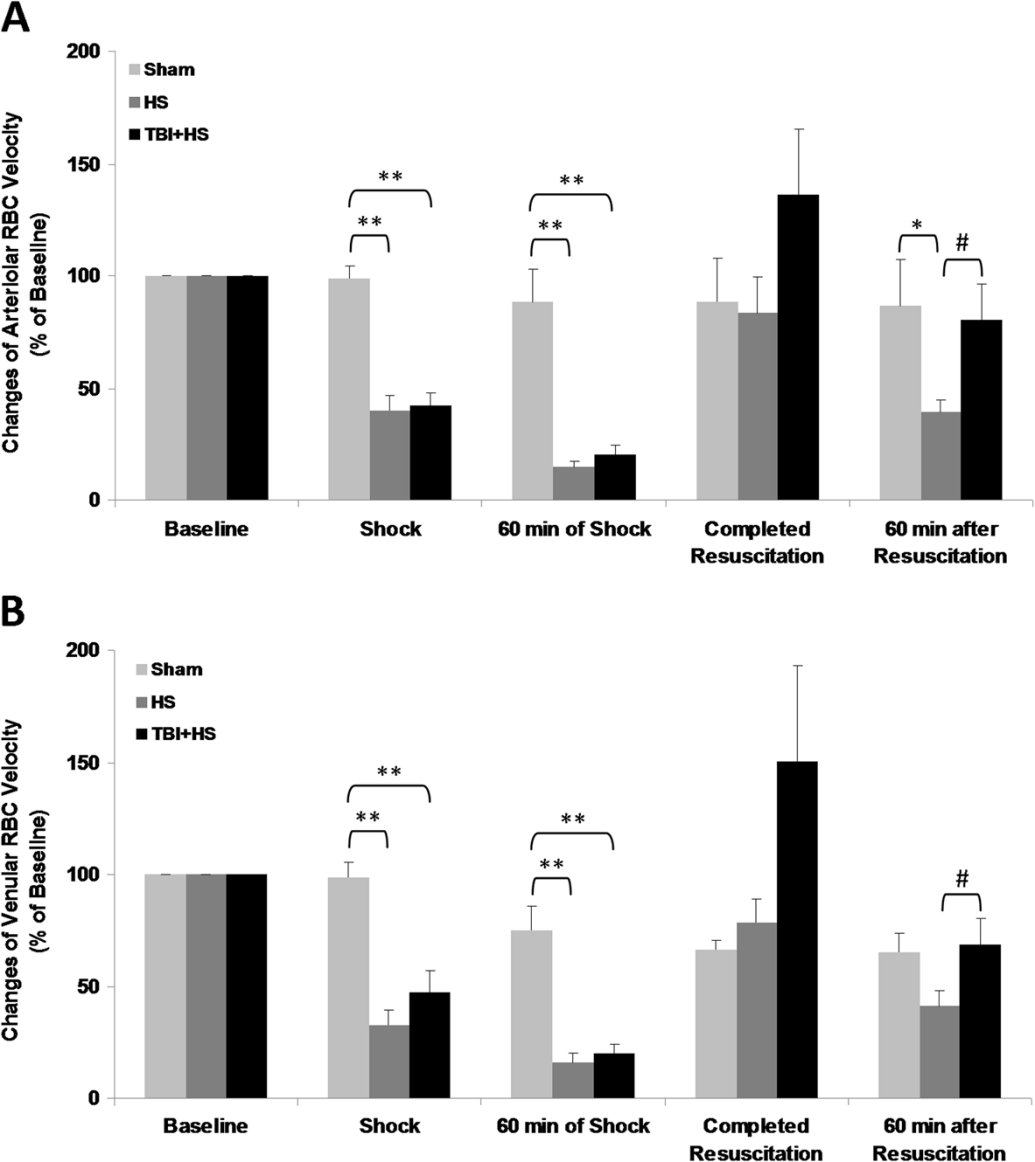
**Red blood cell (RBC) velocity for the three experimental groups and the five observation time points.** The upper panel **(A)** shows arteriolar RBC velocity and the lower panel **(B)** shows venular RBC velocity. (* p < 0.05; ** p < 0.01; # p < 0.05)

**Figure 7:**
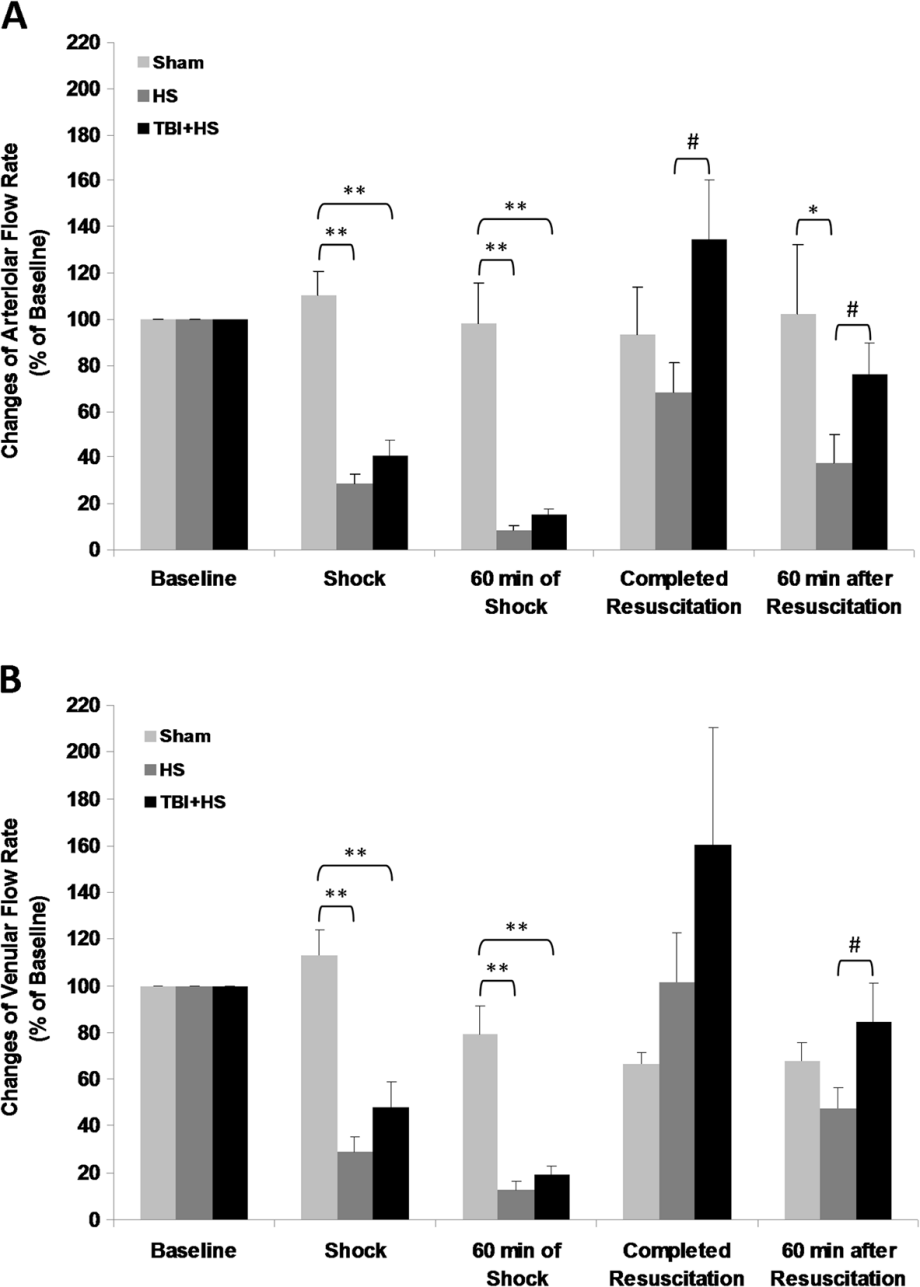
**Blood flow for the three experimental groups and the five observation time points.** The upper panel **(A)** shows arteriolar blood flow and the lower panel **(B)** shows venular blood flow. (* p < 0.05; ** p < 0.01; # p < 0.05)

Arteriolar and venular red-blood-cell (RBC) velocity decreased sharply and progressively to < 20% of baseline during pressure-controlled hemorrhagic shock regardless of presence or absence of superimposed TBI (p < 0.01; Figure 6A and B). After resuscitation, RBC velocity recovered to pre-injury baseline values in HS only animals, whereas in TBI + HS animals RBC velocity was increased to almost 140% of pre-injury baseline values. A comparable pattern was observed when venular RBC velocity was assessed. Interestingly, with 60 minutes of resuscitation RBC velocity in both groups decreased again by > 50% as compared to completed resuscitation reaching approximately 40% of pre-injury baseline in the HS only group and 70% in the HS + TBI group, respectively. Both, arteriolar and venular flow rates followed a similar pattern as shown in Figure 7A and B.

## Discussion

The mortality rates observed in the present study after infliction of both impacts paralleled the mortality rates reported by McMahon and colleagues when using a combined fixed-volume model of hemorrhagic shock combined with TBI in which 50% of all animals died within the first 90 minutes after the completion of hemorrhage [9]. In the clinical scenario, hemorrhagic shock and TBI have frequently been reported to account for approximately 50% of all trauma-related deaths within the first 24 hours after hospital admission [3,4].

Previous reports have indicated that hemorrhagic shock may result in a progressive vasoconstriction of arterioles [15-17]. Accordingly, in the present study acute blood loss during pressure-controlled hemorrhagic shock resulted in a sharp and progressive decrease in the diameter of the third-order arterioles and venules both, at initiation and up to 60 minutes of shock. Interestingly, when TBI was added this constriction was also evident but substantially less pronounced. Obviously, the superimposed TBI counteracted the vasoconstriction from pressure-controlled hemorrhage alone. Law and colleagues have reported similar results when assessing macrocirculatory changes in rats suffering from combined TBI and hemorrhage [13]. These authors could demonstrate that TBI may lead to a reduced ability to modify vascular tone. In detail, brain-injured rats were able to reduce aortal conductance (arterial blood flow/MAP) once hemorrhage was initiated, but they were not able to maintain this vasoconstriction. Furthermore, the response to fluid resuscitation was limited as no relevant changes in aortal conductance were observed. This observation was supported by Yuan and colleagues who demonstrated that TBI may suppress spontaneous hemodynamic recovery from hemorrhage and also impede the efficacy of fluid resuscitation [12]. In contrast to the present study, the studies by Law and colleagues [13] were focused on macrocirculatory changes as reflected by aortic blood flow (ABF) and vascular conductance (ABF/MAP) and on shorter periods of shock, e.g. 30 minutes. The results presented by Yuan and colleagues [12] were focused on cardiovascular responses after TBI and hemorrhage including heart rate, pre- and afterload as well as stroke volumes and indices.

In the present study, resuscitation efforts in shocked but also TBI-injured animals resulted equivocally only in a moderate change in vessel diameter, but simultaneously in a sharp increase in RBC velocity and flow rate. In this context, it is of note that MAP levels were comparable between both groups (HS only and TBI + HS) and therefore could not be made responsible for these microcirculatory changes. Likewise, there were no differences in Hb, pH and PaCO_2_ between the experimental groups observed on repeated blood gas analysis at baseline and during the entire experiment. Therefore, potential acidosis and hypercapnia could also not be made responsible for changes in velocities and vessel diameters.

One possible explanation for the observed changes in vasomotor response and microcirculation may be related to altered sympathetic nervous activity. Traumatic brain injury has been associated with an immediate sympathetic activation as a result of increasing intracranial pressure thus triggering a corresponding increase of circulating catecholamines within minutes after trauma [40-42]. This sympathoadrenal activation with its massive release of vasoactive substances leads to an initial hypertension and an increase in vascular tone. The initial increase in blood pressure shortly after TBI was also observed during our experiments and is confirmed by previous studies [13,18,19].

The idea that there may be sympathetic vasodilatator nerves to skeletal muscles is an old concept fitting with the “fight or flight” model of the sympathetic nervous system [43]. The first evidence for vasodilatator nerves to skeletal muscles emerged when stimulation of skeletal brain stem areas was shown to evoke hypertension, tachycardia and skeletal muscle vasodilatation in terms of the so-called “defense reaction” and that these dilator nerves were cholinergic. Matsukawa and colleagues assessed internal diameter changes of arterial vessels of skeletal muscles evoked by activation of sympathetic cholinergic nerve fibers during stimulation of the hypothalamic defense area in cats. The authors observed a dilatation in small arteries ranging from 100 to 500 μm, but not in larger extramuscular arteries [44]. More recently, the skeletal muscle dilator response to sympathoexitatory maneuvers in both, humans and animals, appears to be nitric oxid (NO)-dependent. Joyner and Diek concluded from their review, that most “sympathetic dilator” responses in human muscle are due to adrenaline or local cholinergic mechanisms acting to stimulate NO release from the vascular endothelium [43].

On the other hand side, Nagai and Pleschka have previously demonstrated circumscribed brain stem sites to mediate adrenergic and non-adrenergic vasoresponses in dogs by electrically probing selected brain stem points to determine from which vasodilatation or vasoconstriction of the lingual and intraorbital arteries could be elicited [45]. Vasodilatation in these vessels by stimulation could be induced from an area extending parasagittally through the ventral part of the brain stem from the hypothalamus to the upper pontine region. The most potent area was in the supraoptic area of the anterior hypothalamus, but no representation was found in the dorsolateral part of the central gray matter, one of the most excitable mesencephalic vasodilatator area in the defense reaction. Centrally elicited vasodilatation of the tongue has been shown to be due to non-adrenergic, non-cholinergic efferents with pre- and postganglionic synapses running apart from the cervical spine trunk. The anatomical origin of these efferentes suggests that they may belong to the parasympathethic section of the autonomic nervous system [45]. It is endogenous to the LFP model to induce bilateral diffuse white matter damage and severely-graded injury to the brain stem including the hypothalamus and pontine regions. In the approach here, the authors have inflicted a TBI of moderate severity and it may be assumed that these triggering areas may have been affected (Figure 1).

The effect of the sympathetic system on vascular tone and microcirculation may also be vessel-diameter dependent. For example, significant hypotension with a mean MAP of 40 mmHg has been shown to induce a reflex response mediated by sympathetic nerves to cause constriction of larger arterioles (70–150 μm) [46]. In small arterioles, however, both, constriction and dilatation, have been observed in response to the same level of hypotension [15,16]. As previously described, Matsukawa and colleagues described dilatation in smaller arteries (100–150 μm), but not in larger extramuscular arteries after hypothalamic defense area stimulation in cats [44]. The underlying mechanism by which hemorrhage induces dilation of smaller arterioles is not fully understood. Humoral factors may play a pivotal role as epinephrine has been shown to induce vasodilatation of small arterioles (<40 μm) by acting through β2-adrenoreceptors [47].

Traumatic brain injury-associated cytokines have also been suggested to promote microcirculatory effects. A whole variety of inflammatory mediators and cytokines are typically released into the systemic circulation after both extra- and intracranial trauma [48-51]. In the cremaster muscle of rats, TNF-α has been shown to modulate arteriolar diameters via an immediate vasodilatory effect [52]. Interestingly, in this experimental setting only third- and fourth-order arterioles were immediately dilated after administration of TNF-α. In contrast, larger vessels did not show any alteration in diameter under this condition [52,53]. It has been speculated that different characteristics of TNF-dependent receptors may mediate this vasodilatation in third-order arterioles, while larger vessels remain unaffected.

Similarly, Interleukin (IL-)1 and IL-6 have frequently been implicated in the decreased systemic vascular resistance in different forms of shock [54]. By also using a cremaster in-vivo preparation in rats, Minghini and colleagues have reported a dose-dependent increase in third-order arteriolar diameters from 11% to 51% with 1 hour in-vivo exposure to IL-1 in increments of 0.01, 0.1, 1.0 to 20 ng/ml. Cytokine washout resulted in arteriolar return to baseline diameter. In vivo exposure to IL-6 (10, 50 and 250 U/ml) for 1 hour yielded similar results, but after washout arteriolar dilatation persisted. These data indicate that both cytokines are potent dilating agents for skeletal muscle resistance vessels under in-vivo conditions.

In the present analysis, resuscitation efforts resulted in an immediate and sharp increase of RBC velocity and arterial flow rate, particularly in TBI + HS animals where an overshooting response to resuscitation was observed. A previous study using the same model of pressure-controlled hemorrhagic shock demonstrated that higher RBC velocities and flow rates after resuscitation were associated with a better survival rate [17]. At first glance, the present results seem contradictory as TBI + HS animals displayed a higher mortality rate despite high arterial blood flow. However, the induced hypoperfusion during the 60 minutes of hemorrhagic shock may result in acute tissue ischemia. It has been shown that the sudden rise in oxygen at the onset of reperfusion is associated with oxidative stress which itself may lead to cellular and organ damage [55,56]. Therefore, a gradual introduction of oxygen during resuscitation seems to be beneficial [57]. It can be speculated, that the overshooting flow rate, as observed in TBI + HS animals, will lead to a faster reperfusion and therefore may be associated with increased organ damage. Nevertheless, it has to be acknowledged that combined animals had sustained a high magnitude of injury in general.

### Limitations

Several limitations of this study have to be acknowledged. In the present analysis, we have focused on selected third-order vessels in a spinotrapezius muscle preparation only. These vessels were selected at random, so we cannot exclude some degree of selection bias. Since we have not assessed other vessel sizes or vascular beds, our results are restricted and interpretation needs caution. The tissue of interest from the critical care perspective is certainly the gastrointestinal tract with secondary complications resulting from mucosal hypoxia and hypoperfusion. Splanchnic ischemia with an intramucosal pH < 7.3 has commonly been reported after isolated TBI [58] and different forms of shock including haemorrhage [59]. Further studies are necessary to analyze the effects of TBI and haemorrhage on the hepatosplanchnic, renal, pulmonary and cardiac microcirculation, all which persist to fail following hemorrhagic shock and may account for the higher overall mortality rate in the combined group.

Furthermore, the present study has focused on the first 60 minutes after resuscitation only. Longer observation periods are warranted to elucidate changes in vessel diameter, RBC velocity and flow rate beyond these time points and to assess chronic effects. Increased levels of vasoactive substances (for example catecholamines, nitric oxide (NO)) as well as trauma-associated circulating mediators and cytokines have been speculated as one possible explanation for the observed differential vasomotor and microcirculatory responses after pressure-controlled hemorrhagic shock with and without superimposed TBI. Further investigations are needed to assess the precise role of these substances in the context of hemorrhagic shock and TBI.

The choice of anesthesia, e.g. sodium pentobarbital, used in the present experiment may have also introduced confounding factors. This agent has been shown to cause metabolic suppression via hypoperfusion and a reduction in cerebral blood flow (CBF) due to flow-metabolism coupling. Pentobarbital has relatively minor effects on cardiac output, arterial pressure and total peripheral resistance, but more important effects on left ventricular function and myocardial contractility [60,61]. For instance, 15 minutes after intravenous pentobarbital, cardiac output, arterial pressure, and total peripheral resistance were all essentially at the pre-anesthetic control levels, but stroke volume was reduced, as was myocardial contractility and the velocity of myocardial fiber shortening [60]. Although this would have affected all investigated animals and the 15 minutes stabilization time may have abolished the effect, it could have been influencing the reactions of those animals subjected to combined TBI and hemorrhage in particular due to the disturbance in circulation caused by the experimental model. Kawaue and Iriuchijima have reported a drop in arterial pressure acutely from an average value of about 105 mmHg to a minimum of about 75 mmHg in about 5 min which then gradually recovered to an average level of about 90 mmHg in 30 min after intravenous injection of pentobarbital sodium [62]. It has to be recognized that pentobarbital in these experiments was administered intervenously while in our study the agent was administered intraperitoneally.

Another limitation of the present analysis is that the experiments have been restricted to the measurement of hemodynamics only. With regard to TBI, behavioural measurements as well as histopathological analyses would have been an interesting addition and will surely be an integral component of our future studies in this field of interest.

Lastly, the findings described here represent our first observations and remain therefore entirely descriptive. The authors are aware that none of the potential mechanisms behind have been explored in detail yet. Additional experiments to close this gap via pharmacological inhibition experiments (for example NO-blocking), cytokine and catecholamine measurements and/or studies in vessels of different diameter are necessary and planned.

## Conclusions

Superimposed TBI modulated arterial and venular responses to hemorrhagic shock in third-order vessels in a spinotrapezius muscle preparation. Overall, third-order vessels in this preparation seemed to lose their ability to respond to changing blood volumes, resulting in a less pronounced vasoconstriction during hemorrhagic shock and an overshoot in RBC velocity and arterial blood flow during and after resuscitation. These results may indicate that TBI diminishes the ability to modulate vascular tone within the microcirculation. Further research is necessary to more precisely assess and define the impact of TBI on the microcirculation in tissues vulnerable to hemorrhagic shock and to further inform the construction of microcirculatory failure as a clinical concept in the critical ill for better outcome [63].

## Abbreviations

AD: Arteriolar diameter; ARRIVE: Animal research: reporting *in vivo* experiments; D: Diameter of the vessel; g: Gram; Hb: Hemoglobin; HS: Hemorrhagic shock; IL: Interleukin; i.p.: Intraperitoneal; IU: International units; kg: Kilogram; LFP: Lateral fluid percussion; MAP: Mean arterial pressure; mg: Milligram; μm: Micrometer; ml: Milliliter; NO: Nitric oxid; RBC: Red blood cell; s: Second; TBI: Traumatic brain injury; TNF: Tumor necrosis factor; U: Unit; V: Velocity; VD: Venular diameter.

## Competing interests

The authors declare that they have no competing interests.

## Authors’ contributions

BC, MMae and QH designed the study. BC and MMae carried out the experiments and analyzed the data obtained. BC and MMu performed the statistical analysis. MMu, QH and MMae drafted the manuscript. EN conceived of the study, participated in its design and coordination, and helped to draft the manuscript. All authors have read and approved the manuscript in its final version prior to submission.
